# Supplementary material for: Short-Term Effects of Side-Stream Smoke on Nerve Growth Factor and Its Receptors TrKA and p75NTR in a Group of Non-Smokers
Source: Int J Environ Res Public Health. 2022 Aug 19;19(16):10317. doi: 10.3390/ijerph191610317 (PMC9408420; doi:10.3390/ijerph191610317)
Supplement: Supplementary file 1 [file ijerph-19-10317-s001.zip › Supplementary Figures.pptx]

## Slide 1
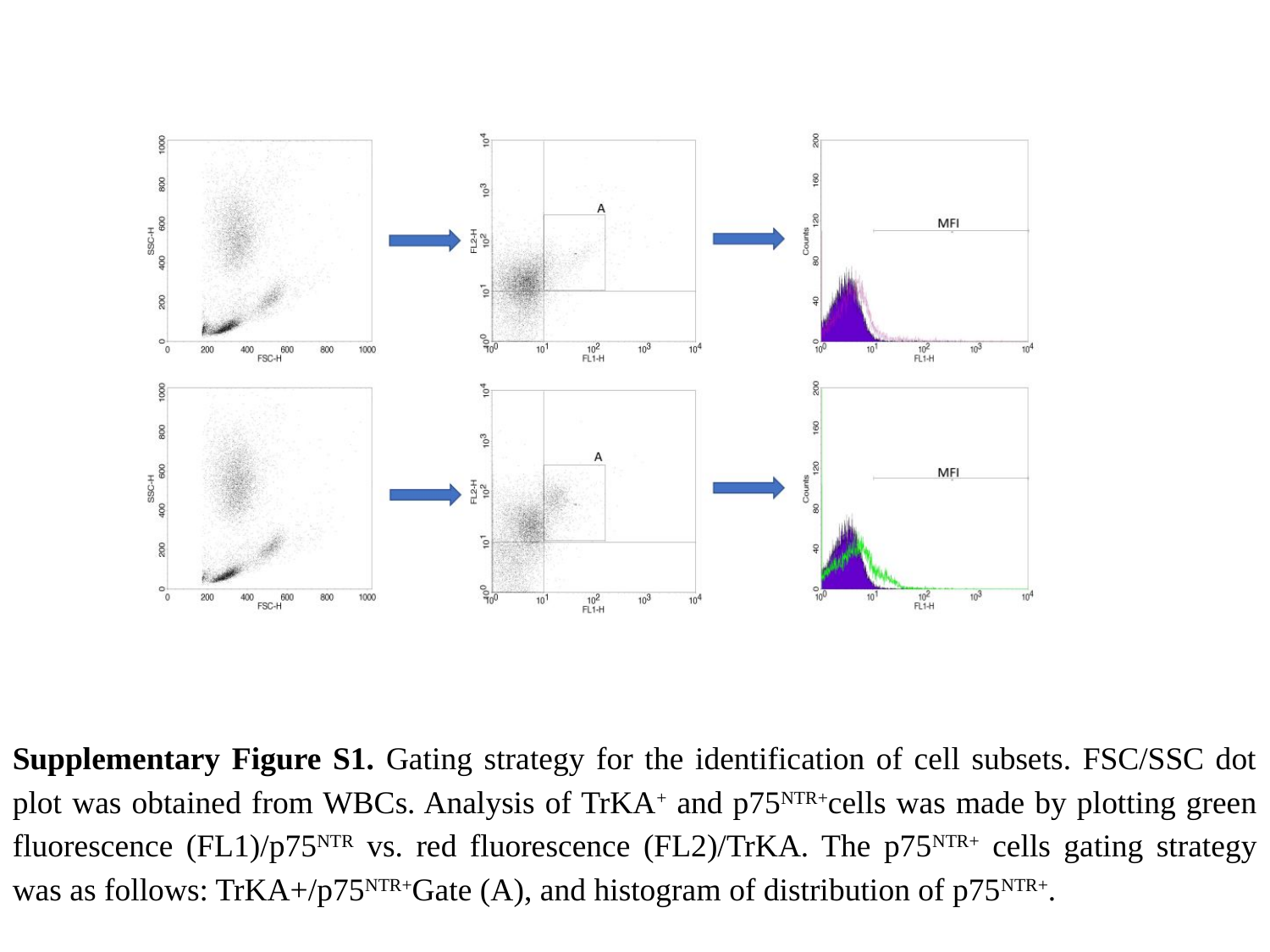

Supplementary Figure S1. Gating strategy for the identification of cell subsets. FSC/SSC dot plot was obtained from WBCs. Analysis of TrKA+ and p75NTR+cells was made by plotting green fluorescence (FL1)/p75NTR vs. red fluorescence (FL2)/TrKA. The p75NTR+ cells gating strategy was as follows: TrKA+/p75NTR+Gate (A), and histogram of distribution of p75NTR+.

## Slide 2
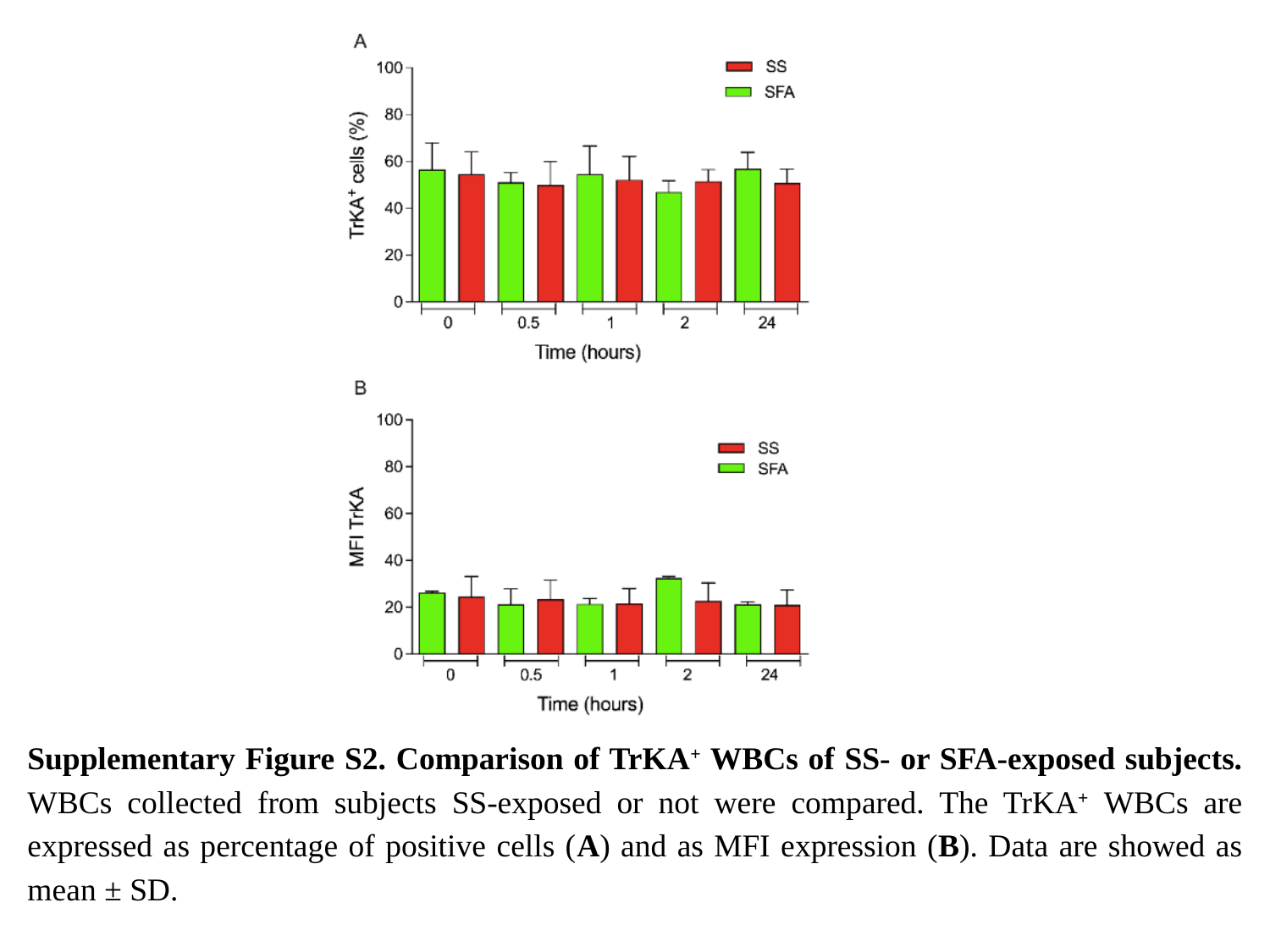

Supplementary Figure S2. Comparison of TrKA+ WBCs of SS- or SFA-exposed subjects. WBCs collected from subjects SS-exposed or not were compared. The TrKA+ WBCs are expressed as percentage of positive cells (A) and as MFI expression (B). Data are showed as mean ± SD.

## Slide 3
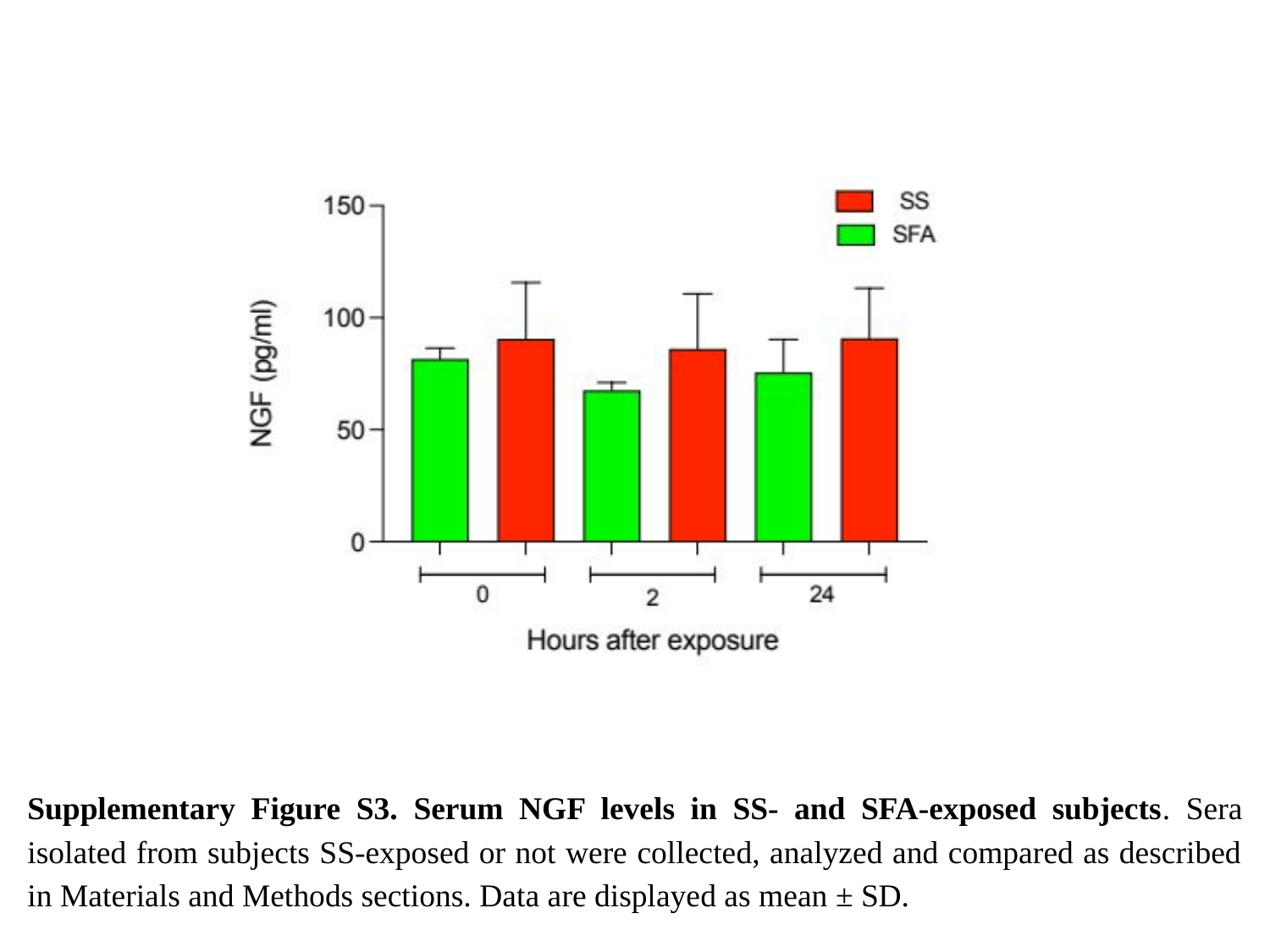

Supplementary Figure S3. Serum NGF levels in SS- and SFA-exposed subjects. Sera isolated from subjects SS-exposed or not were collected, analyzed and compared as described in Materials and Methods sections. Data are displayed as mean ± SD.

## Slide 4
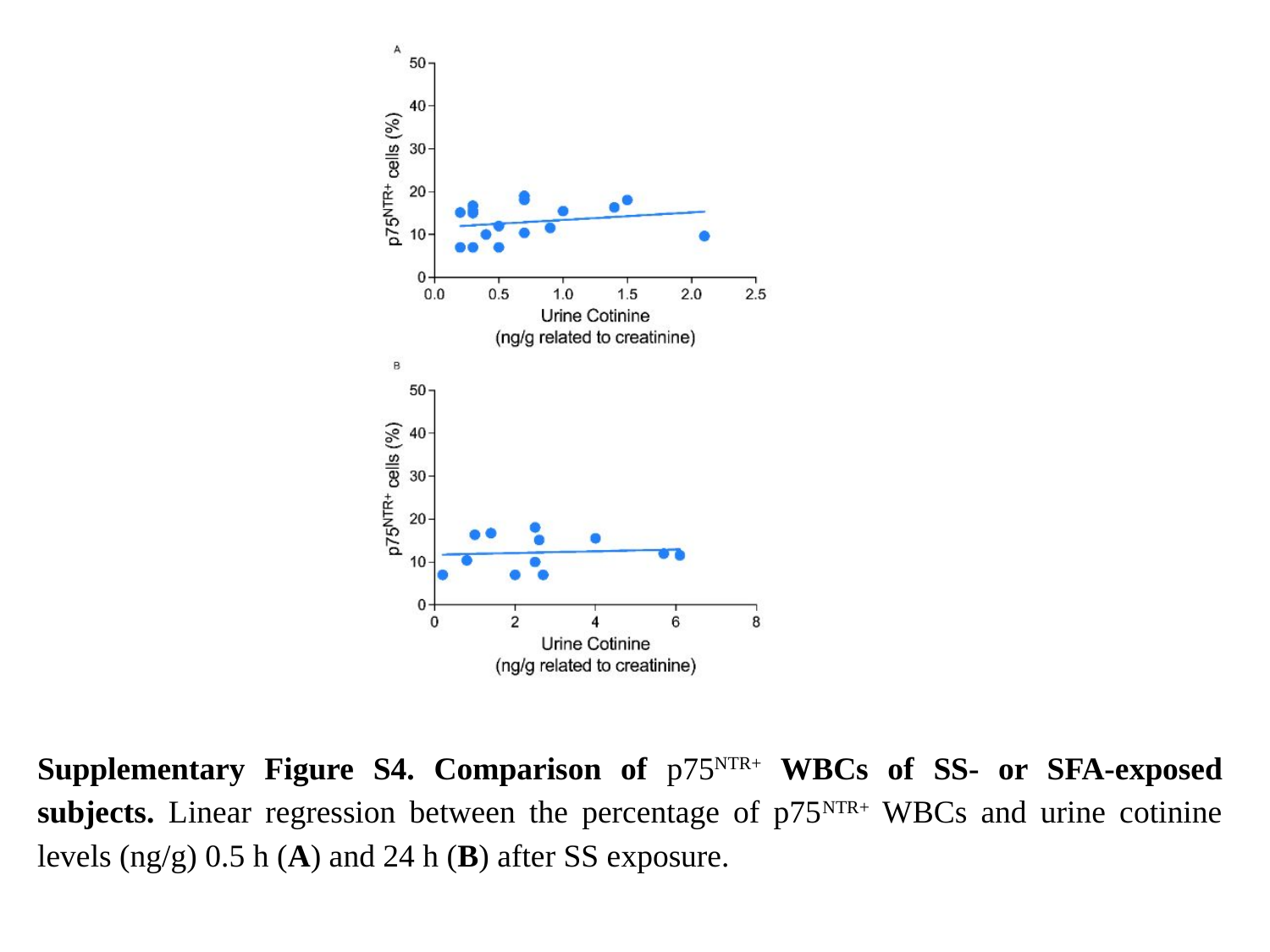

Supplementary Figure S4. Comparison of p75NTR+ WBCs of SS- or SFA-exposed subjects. Linear regression between the percentage of p75NTR+ WBCs and urine cotinine levels (ng/g) 0.5 h (A) and 24 h (B) after SS exposure.

## Slide 5
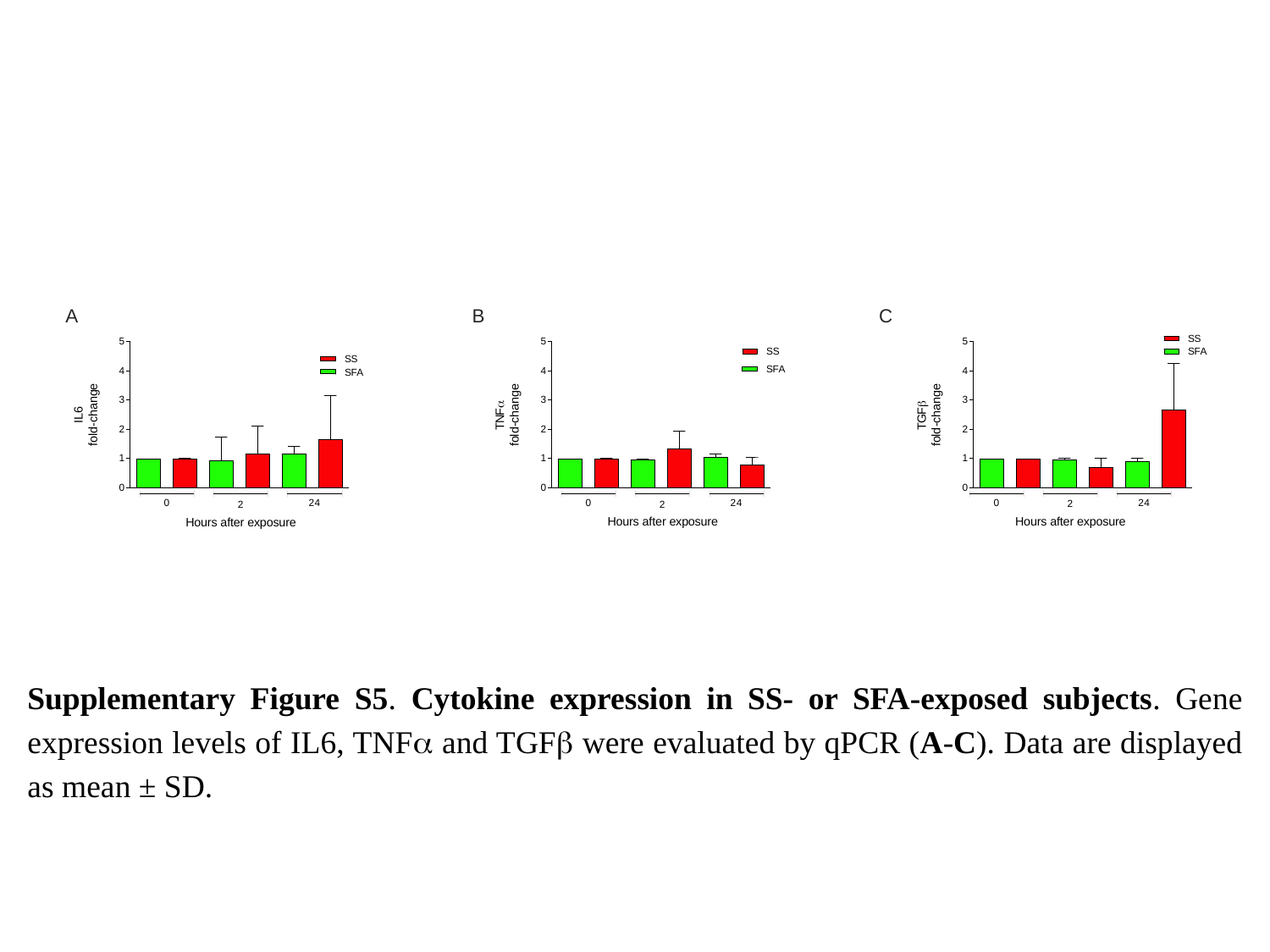

Supplementary Figure S5. Cytokine expression in SS- or SFA-exposed subjects. Gene expression levels of IL6, TNFα and TGFβ were evaluated by qPCR (A-C). Data are displayed as mean ± SD.
