# Supplementary material for: Short-Term Effects of Side-Stream Smoke on Nerve Growth Factor and Its Receptors TrKA and p75NTR in a Group of Non-Smokers
Source: Int J Environ Res Public Health. 2022 Aug 19;19(16):10317. doi: 10.3390/ijerph191610317 (PMC9408420; doi:10.3390/ijerph191610317)
Supplement: Supplementary file 1 [file ijerph-19-10317-s001.zip › Supplementary Table S1.pdf]

Supplementary Table S1. List of primers used in the RT-qPCR analysis. TrKA = Tropomyosin-related kinase A; p75^{NTR} = neurotrophin receptor p75; TNFα = Tumor Necrosis Factor α; TGFβ = Tumor Growth Factor β; IL1β = Interleukin 1β; IL6 = Interleukin 6; GAPDH = Glyceraldehyde-3-phosphate dehydrogenase.

Transcript	Forward Primer 5′-3′	Reverse Primer 5′-3′
TrKA	CAGCCGGCACCGTCTCT	TCCAGGAACTCAGTGAAGATGAAG
p75^{NTR}	GAGGCACCTCCAGAACAAGA	GCTGTTCCACCTCTTGAAGG
TNFα	CTGGGCAGGTCTACTTTGGG	CTGGAGGCCCCAGTTTGAAT
TGFβ	TGGTGGAAACCCACAACGAA	GAGCAACACGGGTTCAGGTA
IL1β	ATGGCAGAAGTACCTAAGCTCGC	ACACAAATGCATGGTGAAGTCAGTT
IL6	CCTGAACCTTCCAAAGATGGC	TTCACCAGGCAAGTCTCCTCA
GAPDH	TCGTGGAAGGACTCATGACC	TCAGCTCAGGGATGACCTTG
β-ACTIN	GCATGGGTCAGAAGGATTC	GTCCCAGTTGGTGACGAT
